# Randomized controlled trial of the effect of phytosterols-enriched low-fat milk on lipid profile in Chinese

**DOI:** 10.1038/srep41084

**Published:** 2017-01-24

**Authors:** Ching-Lung Cheung, Daniel Ka-Chun Ho, Chor-Wing Sing, Man-Fung Tsoi, Vincent Ka-Fai Cheng, Grace Koon-Yee Lee, Yuk-Nam Ho, Bernard M. Y. Cheung

**Affiliations:** 1 Department of Pharmacology and Pharmacy, University of Hong Kong, Pokfulam, Hong Kong; 2 Department of Medicine, University of Hong Kong, Pokfulam, Hong Kong; 3 State Key Laboratory of Pharmaceutical Biotechnology, University of Hong Kong, Pokfulam, Hong Kong

## Abstract

Phytosterols found naturally in plants are known to reduce cholesterol absorption in the gut. The traditional southern Chinese diet typically contains many vegetables and not much meat, and there is high prevalence of lactose intolerance in Chinese; we therefore aimed to test if phytosterols-enriched milk is effective in lowering serum LDL-cholesterol in Chinese. Two hundred and twenty-one participants (41 men and 180 women; age 24–79) without cholesterol-lowering drugs or diabetes mellitus were randomized to daily intake of phytosterols-enriched low-fat milk which contained 1.5 g phytosterols per day (N = 110) or a conventional low-fat milk (N = 111) for three weeks. Fasting bloods were taken before and at the end of the study for the measurement of lipid and glucose profile. Physical examination was also performed. Comparing treatment with control, treatment group had significant decrease in serum LDL-cholesterol level (9.5 ± 2.0%; p < 0.0001). Phytosterols intake also decreased total cholesterol (P < 0.0001) and diastolic blood pressure (P = 0.01). Consumption of a phytosterols-enriched low-fat milk led to a significant fall in LDL-cholesterol, total cholesterol, and diastolic blood pressure in Chinese. This can be recommended as part of a healthy diet for people. (ClinicalTrials.gov identifier: NCT02541201; Date of registration: 26 Aug 2015).

Hypercholesterolemia is the leading cause of coronary heart disease worldwide^1^. Clinical trials as well as meta-analysis have demonstrated that reduction in low-density lipoprotein cholesterol (LDL-C) reduces cardiovascular and all-cause mortality^2^. LDL-C can be reduced by pharmacological and non-pharmacological ways. Taking phytosterols has been suggested as a feasible non-pharmacological way of reducing LDL-C and potentially, also reducing coronary heart disease.

Phytosterols is a collective term that describes a spectrum of plant sterols. They are structurally and functionally similar to animal cholesterol, but cannot be utilized by humans and compete with cholesterol absorption in the gut. Thus, it inhibits intestinal absorption of cholesterol and thereby lowers blood cholesterol levels. The role of phytosterols in lowering LDL-C has been well recognized by health authorities worldwide, including EU, the US, Canada, and Australia/New Zealand^3^. Recently, FDA has gone further than previously and approved the health claim “Foods containing at least 0.65 gram per serving of vegetable oil plant sterol esters, eaten twice a day with meals for a daily total intake of at least 1.3 grams, as part of a diet low in saturated fat and cholesterol, may reduce the risk of heart disease”^4^.

Phytosterols are naturally found in vegetables. Traditional southern Chinese diet is characterized by high intakes of vegetables and moderate intakes of animal foods^5^. Thus, it remains largely unknown if additional intake of phytosterols may help to reduce LDL-C in people with high dietary vegetable intakes. Dairy product is the most commonly used food carrier for phytosterols-enriched food. Given that there is high prevalence of lactose intolerance in Chinese, the effectiveness of phytosterols-enriched milk in reducing LDL-C in Chinese is uncertain. We hypothesized that consuming phytosterols-enriched low fat milk would reduce LDL-C in Chinese. To test this hypothesis, we conducted a double-blind, randomized, placebo-controlled trial to study the effect of phytosterols-enriched low fat milk on serum lipid profile.

## Materials and Methods

The study was a randomized, double-blinded, single-center, two-arm, placebo-controlled phase 2 clinical trial that examined the effect of consuming a plant-sterols-enriched low-fat milk for 3 weeks on blood lipids. We conducted this trial in a tertiary institution-based hospital in Hong Kong. Subjects were healthy Southern Chinese male or female aged ≥ 18 years recruited from the community. Subjects were excluded if they met any of the following criteria: familial hypercholesterolemia; ingestion of drugs known to interfere with lipid profiles (e.g. hormone replacement treatment, diuretics, beta-blockers, statin or other hypocholesterolemic drug treatment); smoking > 10 cigarettes/day; heavy alcohol intake (>40 g/day for men; >30 g/day for women); milk intolerance or dislike, or known allergy or hypersensitivity to milk proteins, soy and peanut; major chronic diseases such as diabetes (type I or type II), renal or liver disease; refusing to stop the consumption of plant-sterols-enriched products other than the study product, if any, during the study; receiving systemic treatment or topical treatment likely to interfere with the evaluation of the study parameters; pregnancy or lactation during the study period. Briefly, each participant was randomly assigned to one of two groups and given milk powder containing phytosterols at a daily dose of either 0 g (placebo group with phytosterols 0 g/d; n = 111) or 1.5 g (treatment group with phytosterols 1.5 g/d; n = 110), for a period of 3 weeks in a double-blind manner. A web-based randomization system was used for online group allocation. Block randomization was used to maintain approximately 1:1 allocations into the two study groups. Investigators and participants were blinded during the study period. The double-blind coding was not revealed until data analysis. The study was approved by the Institutional Review Board of the University of Hong Kong/Hospital Authority Hong Kong West Cluster (HKU/HA HKW IRB). This study was conducted in accordance to ICH-GCP guideline. Participants gave written informed consent before participation. The trial was registered at clinicaltrials.gov number NCT02541201 on 25 Aug 2015. The trial ended as planned in the protocol.

### Demographic information, medical and medication history

Demographic information including age and sex were collected during the baseline visit. Medical history including medications history, concomitant treatments, alcohol and tobacco use, were obtained during the screening and baseline visit, and supplemented by medical records if necessary. Complete medications history of prescription and over-the-counter medications within 3 months before the screening and baseline visit was collected.

### Study product and dietary intervention

The product used in the experimental group was a dried partially-skimmed milk powder with no more than 12% mild fat (11.4 g total fat/100 g product) containing unesterified, unhydrogenated plant sterols. The tested milk product is commercially known as OMEGA PLUS milk (or Double care phytosterol nutritional milk in Hong Kong)^6^, and the bioactivity of phytosterols is preserved during the production of the milk powder according to the manufacturer. The product used in the control group was the same low-fat milk powder but containing no plant sterols. Participants in the experimental group were planned to consume orally 1.5 g plant sterols daily provided in two servings, each serving taken immediately after breakfast and lunch, of the milk product for three consecutive weeks. One serving consisted of 246 ml warm water (40–50 °C) and 38.1 g milk powder that made up 273 ml of the final milk product. Participants in the control group consumed the placebo low-fat milk with the same administration regimen and schedule. Both groups were advised not to change their dietary habits during the study period.

### Study compliance

The study product was given to the subject during their screening and baseline visit (Day 0). At the end of the study intervention period (Day 21 ± 5), subjects were required to return all unused product to measure compliance. At the same time, each participant was given a card for recording “how many times that the study product was taken each day”.

### Physical and laboratory assessments

Physical assessments were performed at baseline and at the end of the study. These assessments included blood pressure, body temperature, body height and weight, and waist and hip circumference. Blood pressure was measured using a GE CARESCAPE V100 (Fairfield, CT) automated blood pressure monitor, waist and hip circumference were measured at the midpoint between the last rib and the iliac crest, and at the level of the largest lateral extension of the hips, respectively, in a horizontal plane. Participants were advised to fast for at least 8 hours prior to the visit. Serum LDL-C, high-density lipoprotein cholesterol (HDL-C), total cholesterol, triglycerides, creatinine, and fasting blood glucose were measured using the Ortho-Vitros Fusion 5.1 automated analyzer at baseline and at the end of the study.

### Statistical analysis

Sample size of this study was calculated by using PASS 12. ANCOVA was used to estimate the sample size with serum LDL-C of baseline as independent covariate, minimum power of 0.8 according to the previous study^6^, attrition rate of 15% and 5% of the maximum tolerable false positive rate.

For continuous variables, results were expressed as mean and standard deviation. For categorical variables, the number and percentage of participants were reported. The intention-to-treat (ITT) population consisted of all cases that had been randomized. The per-protocol (PP) population included all eligible participants who had: 75% study product compliance, primary efficacy data collected within the pre-specified allowable visit window, or no protocol violation that was clinically significant.

The primary objective was to compare the serum LDL-C level at week 3 between the groups. ANCOVA was used to evaluate the association in terms of least square (LS) mean difference and % change. Baseline LDL-C was adjusted as a covariate. The secondary objective was to compare other cardiometabolic risk factors (blood pressure, BMI, waist and hip circumferences, serum HDL-C, total cholesterol, triglycerides, and fasting glucose) between the groups. The primary and secondary efficacy analyses were performed on the intention-to-treat (ITT) population.

Subgroup analyses were performed to make comparison of LS mean change of primary and secondary endpoints between groups in the PP population; and change in serum lipid profile of subjects in ITT population stratified by baseline LDL-C level between groups.

## Results

**Figure 1:**
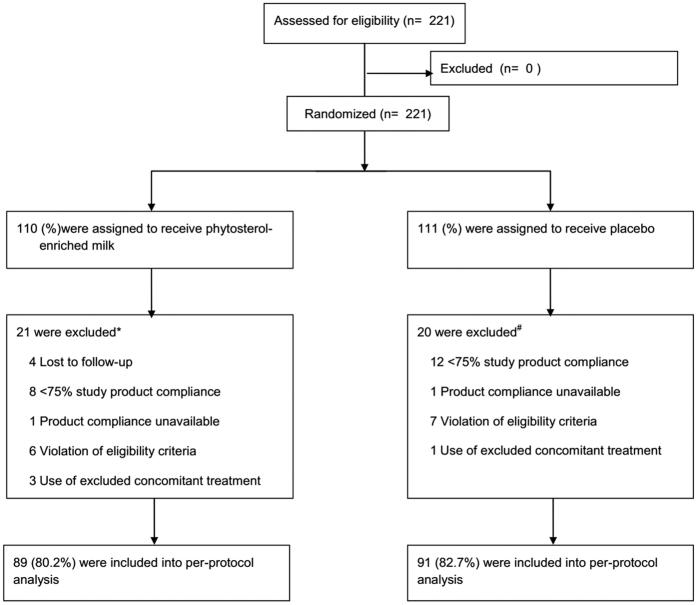
Patient enrolment, randomisation and follow-up. ^*^1 patient was excluded due to both violation of eligibility criteria and lost to follow-up. ^#^1 patient was excluded due to both violation of eligibility criteria and <75% study product compliance.

A total of 221 subjects were recruited (110 in the treatment group and 111 in the control group) in July 2015 and follow-up was completed in mid-September 2015 (Fig. 1). There were no statistically significant differences of baseline characteristics between groups (Table 1).

No significant difference in product intake between groups was observed. During the 21-day study period, 8 and 12 from treatment and placebo group had product compliance ≤75%. Product compliance data were unavailable in one participant from each group. The difference in treatment adherence was not statistically significant.

After three weeks’ intervention, four participants in the treatment group failed to return to the final visit. Subjects who received phytosterols-enriched milk had a reduction in serum LDL-C by LS mean (standard error [SE]) of 0.265 mmol/L (SE, 0.056) (Table 2) when compared with the placebo and after adjusted for baseline LDL-C. The LDL-C was reduced by 4.58% (SE, 1.45) in the experimental group while that in the placebo group it increased by 4.88% (SE, 1.42). Similar results were obtained in the per-protocol population (Supplementary Table 1) and no sex interaction was found (data not shown). In subgroup analyses stratified by the LDL levels, similar results were obtained except that a larger effect was observed in participants with high LDL levels (Supplementary Table 2).

Among secondary endpoints, taking phytosterols-enriched milk resulted in a significant decrease in total cholesterol by 0.262 mmol/L (SE, 0.06; P < 0.0001) and diastolic blood pressure by 1.93 mmHg (SE, 0.746; P = 0.01) when compared to placebo (Table 2). We did not find any significant change in systolic blood pressure, height, weight, BMI, waist and hip circumferences, serum creatinine, HDL-C, triglycerides, and fasting glucose levels after supplementation. Similar results were observed in the PP population (Supplementary Table 1). In subgroup analyses stratified by the LDL levels, the beneficial effect of phytosterols on total cholesterol was only observed in people with normal or marginal LDL levels; whereas no effect was observed between phytosterols and diastolic blood pressure (Supplementary Table 2).

Similar percentages of patients from treatment (27.3%) and placebo (32.4%) groups had at least one adverse event (Table 3). The frequency of adverse events was not significantly different between groups. The most common adverse event was diarrhea, followed by flatulence.

## Discussion

In the present study, we examined the effects of phytosterols on LDL-C and other cardiometabolic risk factors in Chinese. The main finding was that consumption of 1.5 g phytosterols per day reduced LDL-C, total cholesterol, and diastolic blood pressure.

The beneficial effect of phytosterols on reduction of LDL-C and total cholesterol has been well documented in Caucasian populations. Our findings in the Chinese are concordant with those reported in other populations, showing that daily intake of phytosterols is useful in reducing LDL-C and total cholesterol. There has been a report of the effect of phytosterols in Chinese. A double-blind, randomized trial was conducted in 309 participants to investigate the effect of plant sterol-enriched milk tea on blood lipids after 5 weeks consumption. Interestingly, 2.3 g/d and 1.5 g/d plant sterol reduced serum total cholesterol by 0.25 and 0.23 mmol/l, respectively, when compared with placebo. However, the effect of both doses on LDL-C was not statistically significant^7^.

Consumption of phytosterols may have beneficial effect on blood pressure. Previous animal study showed that consumption of phytosterols-rich diet increased systolic and diastolic blood pressure in salt-loaded stroke-prone spontaneously hypertensive^8^ and non-salt-loaded normotensive Wistar–Kyoto^9^ inbred rats when compared to rats consuming the control diet. However, it should be noted that the phytosterols diet led to ~4 fold increase in serum phytosterols when compared to control diet in rat^9^, whereas the effect of consuming 1.5 g phytosterols per day for 3 weeks is expected to cause <15% increase in serum phytosterols in humans^10^. In contrast, human trials related to phytosterols and blood pressure yielded inconsistent results. In general, blood pressure was a secondary outcome in these trials and no direct effect was observed from phytosterols supplementation on blood pressure, except in one trial. In the trial investigating the effect of bioactive milk peptides and phytosterols on cardiovascular risk factors in 104 hypertensive and hypercholesterolemic subjects, daily intake of spread containing 4.2 mg bioactive milk peptides and 2 g phytosterols for 10 weeks led to reduction in systolic blood pressure in the treatment group^11^. However, it is not possible to infer if the blood pressure lowering effect is from bioactive milk peptides, phytosterols, or their interaction. In the current study, we observed that diastolic blood pressure did not significantly change in the phytosterol group but increased significantly by 2 mmHg in the control group. As this was not a primary endpoint, no strong conclusions can be drawn. However, it raises the possibility that phytosterols have a beneficial effect on diastolic blood pressure, but this hypothesis requires confirmation in future studies.

Chinese are known to have a high prevalence of lactose intolerance when compared to Caucasians, so it is possible that using milk powder as a carrier of phytosterols may reduce its LDL-C lowering effect. In the current study, around 30% of participants experienced adverse events, and the most common adverse event was diarrhea. Interestingly, the most common adverse event of taking phytosterols-enriched low fat milk was constipation in Caucasians^6^. Despite the high incidence of diarrhea, the effect of phytosterols on LDL-C was comparable to those observed in Caucasians^6^. Moreover, our analysis did not reveal any significant interaction between the degree of LDL-C reduction and the presence of adverse effect. Furthermore, a similar association was observed in subgroup analysis in participants with adverse effects (data not shown). Thus, phytosterols-containing low-fat milk is effective in lowering LDL-C even in people with adverse effects.

Although statins remain the first-line class of drugs to lower LDL-C, there are advantages in using a non-pharmacological intervention, albeit less efficacious. Statins may cause myositis and liver enzyme elevations in a small percentage of patients. They are now known to increase the risk of developing diabetes^12^. In contrast, phytosterols are well-tolerated and serious adverse effects are almost absent^13^. This makes phytosterols the intervention of choice in those at low cardiovascular risk and have no or only mild elevation in LDL-C. In patients who are already prescribed statins, phytosterols can enhance the lipid lowering effect of statins by acting on a different mechanism, akin to the rational combination of statin and ezetimibe. This suggestion has been confirmed by a recent meta-analysis, which showed that phytosterols-enriched diet further lower LCL-C in patients treated with statins^14^.

Low calcium intake and osteoporosis are common in Asian populations^15^. Our study showed that phytosterols-enriched low-fat milk was well tolerated in a large proportion of Chinese and this type of drink not only lowers cholesterol and blood pressure, but also provides an important daily source of calcium and protein.

A potential implication of our findings is that consuming 1.5 g of phytosterols every day reduces LDL-C, regardless of whether the baseline LDL-C is normal, marginal or high. Nevertheless, there were limitations in the current study. First, study participants may be more motivated and compliant than the general population. Moreover, compliance may be different in the long term. Second, we have no detailed information on the diet before and during the trial, so we cannot be sure that there were no unintentional changes in the diet during the study. Nevertheless, there was no fall in LDL-C in the control group. Moreover, it has been shown that LDL-C lowering effect of phytosterols is not related to dietary cholesterol or saturated fat intake^16^. Third, we did not measure plasma phytosterols and lathosterol levels, therefore we could not evaluate effect of supplementing phytosterols on their serum level. Fourth, since we have no detailed information on the diet before and during the trial, we could not evaluate the dietary intake of phytosterols in the diet of the participants. Thus, phytosterols in the diet could also have contributed to the lowering of LDL-C, although very few foods contain as much phytosterols as the phytosterol-enriched milk we tested.

In conclusion, phytosterols-enriched milk consumption with 1.5 g phytosterols per day is an effective way to reduce LDL-C in people with normal, marginal, or high LDL-C levels.

## Additional Information

**How to cite this article:** Cheung, C.-L. *et al*. Randomized controlled trial of the effect of phytosterols-enriched low-fat milk on lipid profile in Chinese. *Sci. Rep.* **7**, 41084; doi: 10.1038/srep41084 (2017).

**Publisher's note:** Springer Nature remains neutral with regard to jurisdictional claims in published maps and institutional affiliations.

## Supplementary Material

Supplementary Information

## Figures and Tables

**Table 1:** Baseline characteristics of the study participants.

	Treatment (N = 110)	Placebo (N = 111)	p-value
Age (years)	54.09 ± 11.012	52.93 ± 10.877	0.432
Female	91 (82.73%)	89 (80.18%)	
Height (cm)	158.95 ± 7.26	159.24 ± 8.487	0.801
Weight (kg)	56.91 ± 10.331	57.25 ± 9.38	0.786
BMI (kg/m^2^)	22.45 ± 3.371	22.52 ± 3.018	0.86
Body temperature (°C)	36.47 ± 0.304	36.5 ± 0.287	0.331
Waist circumference (cm)	80.64 ± 8.843	81.83 ± 8.543	0.322
Hip circumference (cm)	95.48 ± 6.696	95.21 ± 5.834	0.747
LDL-C (mmol/L)	3.22 ± 0.818	3.08 ± 0.779	0.197
HDL-C (mmol/L)	1.53 ± 0.4	1.5 ± 0.383	0.547
Total cholesterol (mmol/L)	5.25 ± 0.801	5.05 ± 0.682	0.056
Triglycerides	1.21 ± 0.6	1.15 ± 0.478	0.439
Creatinine (μmol/L)	65.53 ± 12.239	64.34 ± 11.593	
Fasting glucose (mmol/L)	4.95 ± 0.738	4.86 ± 0.464	0.301
Systolic blood pressure (mmHg)	123.35 ± 17.016	122.26 ± 16.473	0.628
Diastolic blood pressure (mmHg)	78.86 ± 10.576	77.73 ± 9.683	0.407

Values are given as no. (%) or mean ± SD.

**Table 2:** LS mean change of clinical endpoints after the intervention in the ITT population.

Variable	Treatment (N = 110)	Placebo (N = 111)	Treatment effect	P-value
	Mean change (95% CI)	Mean change (95% CI)	Mean difference (95% CI)	
LDL-C (mmol/L)	−0.154 (−0.233, −0.076)	0.111 (0.034, 0.188)	−0.265 (−0.375, −0.155)	<0.0001
HDL-C (mmol/L)	−0.055 (−0.082, −0.027)	−0.072 (−0.099, −0.045)	0.017 (−0.022, 0.055)	0.395
Total cholesterol (mmol/L)	−0.236 (−0.32, −0.151)	0.026 (−0.057, 0.109)	−0.262 (−0.38, −0.143)	<0.0001
Triglycerides (mmol/L)	0.066 (−0.022, 0.155)	0.093 (0.006, 0.179)	−0.026 (−0.15, 0.097)	0.674
Creatinine (μmol/L)	1.229 (0.438, 2.021)	1.906 (1.13, 2.683)	−0.677 (−1.786, 0.433)	0.231
Fasting glucose (mmol/L)	0.019 (−0.048, 0.085)	−0.023 (−0.088, 0.042)	0.042 (−0.051, 0.135)	0.379
Systolic blood pressure (mmHg)	−2.749 (−4.4, −1.097)	−1.438 (−3.052, 0.176)	−1.311 (−3.62, 0.999)	0.265
Diastolic blood pressure (mmHg)	0.177 (−0.875, 1.229)	2.11 (1.083, 3.138)	−1.934 (−3.405, −0.462)	0.01
Body temperature (°C)	−0.037 (−0.075, 0.001)	−0.018 (−0.054, 0.019)	−0.019 (−0.072, 0.033)	0.469
Weight (kg)	0.311 (0.009, 0.613)	0.028 (−0.268, 0.323)	0.284 (−0.139, 0.706)	0.187
Height (cm)	−0.5 (−0.708, −0.292)	−0.405 (−0.609, −0.202)	−0.095 (−0.386, 0.196)	0.522
BMI (kg/m^2^)	0.262 (0.129, 0.395)	0.13 (0, 0.26)	0.132 (−0.054, 0.317)	0.165
Waist circumference (cm)	−0.699 (−1.040, −0.359)	−0.669 (−0.998, −0.340)	−0.031 (−0.504, 0.443)	0.899
Hip circumference (cm)	−0.235 (−0.733, 0.264)	−0.43 (−0.907, 0.047)	0.195 (−0.495, 0.885)	0.577

Baseline value was adjusted in the model.

**Table 3:** Treatment-related adverse events.

Events	Treatment (N = 110)	Placebo (N = 111)	P-value
Subjects with at least one adverse event	30 (27.3)	36 (32.4)	0.463
Most common adverse events (>1%)			
Diarrhea	18 (16.4)	21 (18.9)	0.725
Mild	16	16	
Moderate	1	4	
Severe	1	1	
Flatulence	14 (12.7)	18 (16.2)	0.567
Mild	10	14	
Moderate	4	4	
Nausea	1 (0.9)	3 (2.7)	0.622
Mild	1	3	
Abdominal cramps or stomachache	0 (0)	3 (2.7)	0.247
Moderate	0	2	
Severe	0	1	
Weight gain	4 (3.6)	0 (0)	0.06
Mild	4	0	
Weight loss	2 (1.8)	2 (1.8)	1

Data are presented as N (%).

## References

[b1] Yusuf S. . Effect of potentially modifiable risk factors associated with myocardial infarction in 52 countries (the INTERHEART study): case-control study. Lancet. 364 (2004). 10.1016/S0140-6736(04)17018-9 15364185

[b2] Cheung B. M., Lauder I. J., Lau C. P. & Kumana C. R. Meta-analysis of large randomized controlled trials to evaluate the impact of statins on cardiovascular outcomes. Br J Clin Pharmacol 57, 640–651 (2004). 15089818 10.1111/j.1365-2125.2003.02060.x PMC1884492

[b3] Jew S. . Nutrient essentiality revisited. Journal of Functional Foods 14, 203–209 (2015).

[b4] *CFR* - *Code of Federal Regulations Title 21*, https://www.accessdata.fda.gov/scripts/cdrh/cfdocs/cfcfr/CFRSearch.cfm?fr=101.83 (2015).

[b5] Li Y. . Dietary patterns are associated with stroke in Chinese adults. The Journal of nutrition 141, 1834–1839 (2011). 21865562 10.3945/jn.111.143883

[b6] Thomsen A. B., Hansen H. B., Christiansen C., Green H. & Berger A. Effect of free plant sterols in low-fat milk on serum lipid profile in hypercholesterolemic subjects. Eur J Clin Nutr. 58, 860–870 (2004). 15164106 10.1038/sj.ejcn.1601887

[b7] Li N. Y. . Plant sterol-enriched milk tea decreases blood cholesterol concentrations in Chinese adults: a randomised controlled trial. Br J Nutr 98, 978–983 (2007). 17617940 10.1017/S0007114507754302

[b8] Chen Q. . Influence of dietary phytosterols and phytostanols on diastolic blood pressure and the expression of blood pressure regulatory genes in SHRSP and WKY inbred rats. Br J Nutr 102, 93–101 (2009). 19025722 10.1017/S0007114508137904

[b9] Chen Q. . Dietary phytosterols and phytostanols decrease cholesterol levels but increase blood pressure in WKY inbred rats in the absence of salt-loading. Nutr Metab (Lond) 7, 11 (2010). 20637058 10.1186/1743-7075-7-11 PMC2843689

[b10] Tuomilehto J. . Safety assessment of common foods enriched with natural nonesterified plant sterols. Eur J Clin Nutr 63, 684–691 (2009). 18270526 10.1038/ejcn.2008.11

[b11] Turpeinen A. M. . A spread containing bioactive milk peptides Ile-Pro-Pro and Val-Pro-Pro, and plant sterols has antihypertensive and cholesterol-lowering effects. Food Funct 3, 621–627 (2012). 22398753 10.1039/c2fo10286b

[b12] Sattar N. . Statins and risk of incident diabetes: a collaborative meta-analysis of randomised statin trials. Lancet 375, 735–742 (2010). 20167359 10.1016/S0140-6736(09)61965-6

[b13] QuIlez J., GarcIa-Lorda P. & Salas-Salvado J. Potential uses and benefits of phytosterols in diet: present situation and future directions. Clin Nutr 22, 343–351 (2003). 12880600 10.1016/s0261-5614(03)00060-8

[b14] Han S. . Effects of plant stanol or sterol-enriched diets on lipid profiles in patients treated with statins: systematic review and meta-analysis. Scientific reports. 6, 31337 (2016). 27539156 10.1038/srep31337 PMC4990897

[b15] Kung A. W., Lee K. K., Ho A. Y., Tang G. & Luk K. D. Ten-year risk of osteoporotic fractures in postmenopausal Chinese women according to clinical risk factors and BMD T-scores: a prospective study. J Bone Miner Res 22, 1080–1087 (2007). 17371165 10.1359/jbmr.070320

[b16] Hernandez-Mijares A. . Effects of phytosterol ester-enriched low-fat milk on serum lipoprotein profile in mildly hypercholesterolaemic patients are not related to dietary cholesterol or saturated fat intake. Br J Nutr 104, 1018–1025 (2010). 20456813 10.1017/S0007114510001686

