# Supplementary material for: Randomized controlled trial of the effect of phytosterols-enriched low-fat milk on lipid profile in Chinese
Source: Sci Rep. 2017 Jan 24;7:41084. doi: 10.1038/srep41084 (PMC5259797; doi:10.1038/srep41084)
Supplement: Supplementary Information [file srep41084-s1.pdf]

Supplementary Information for Randomized controlled trial of the effect of phytosterols-enriched low-fat milk on lipid profile in Chinese

Title of manuscript:

Randomized controlled trial of the effect of phytosterols-enriched low-fat milk on lipid profile in Chinese

Ching-Lung Cheung[1,2,3], Daniel Ka-Chun Ho[1,2], Chor-Wing Sing[1], Man-Fung Tsoi[2], Vincent Ka-Fai Cheng[1], Grace Koon-Yee Lee[1], Yuk-Nam Ho[1], Bernard MY Cheung[2,3].

[1]Department of Pharmacology and Pharmacy

[2]Department of Medicine

[3]State Key Laboratory of Pharmaceutical Biotechnology

University of Hong Kong, Pokfulam, Hong Kong

Supplementary table 1. LS mean change of primary and secondary endpoints after the intervention in the PP population.

Variable	Treatment (N=89)		Placebo (N=91)		Treatment effect		P-value
	Mean change (95% CI)		Mean change (95% CI)		Mean difference (95% CI)		
LDL-C (mmol/L)	-0.181	(-0.267, -0.095)	0.095	(0.009, 0.18)	-0.275	(-0.397, -0.154)	<0.0001
HDL-C (mmol/L)	-0.053	(-0.083, -0.024)	-0.078	(-0.107, -0.049)	0.025	(-0.017, 0.066)	0.24
Total cholesterol (mmol/L)	-0.262	(-0.354, -0.17)	-0.001	(-0.093, 0.091)	-0.261	(-0.392, -0.131)	<0.0001
Triglycerides (mmol/L)	0.054	(-0.043, 0.15)	0.085	(-0.011, 0.181)	-0.031	(-0.168, 0.105)	0.652
Creatinine (µmol/L)	1.232	(0.375, 2.089)	1.715	(0.862, 2.567)	-0.482	(-1.691, 0.727)	0.432
Fasting glucose (mmol/L)	-0.022	(-0.075, 0.03)	-0.038	(-0.09, 0.014)	0.016	(-0.059, 0.09)	0.675
Systolic blood pressure (mmHg)	-3.635	(-5.397, -1.873)	-1.5	(-3.242, 0.243)	-2.136	(-4.615, 0.343)	0.091
Diastolic blood pressure (mmHg)	-0.077	(-1.233, 1.078)	2.197	(1.054, 3.339)	-2.274	(-3.899, -0.649)	0.006
Body temperature (°C)	-0.048	(-0.088, -0.007)	-0.021	(-0.061, 0.019)	-0.027	(-0.083, 0.03)	0.352
Weight (kg)	0.409	(0.075, 0.744)	0.053	(-0.278, 0.384)	0.356	(-0.114, 0.827)	0.137
Height (cm)	-0.483	(-0.713, -0.254)	-0.472	(-0.699, -0.245)	-0.011	(-0.334, 0.312)	0.945
BMI (kg/m^2)	0.298	(0.147, 0.449)	0.156	(0.007, 0.306)	0.141	(-0.071, 0.354)	0.191
Waist circumference (cm)	-0.064	(-0.588, 0.459)	-0.412	(-0.915, 0.09)	0.348	(-0.38, 1.076)	0.346
Hip circumference (cm)	-0.53	(-0.911, -0.149)	-0.614	(-0.987, -0.242)	0.084	(-0.448, 0.617)	0.755

Baseline value was adjusted in the model

Supplementary table 2. Effects of phytosterols compared with placebo on serum lipid profile in people with different LDL-levels (ITT population).

Lipid variables	LDL-levels	Treatment (N=110) Mean change (95% CI)	Placebo (N=111) Mean change (95% CI)	Treatment effect Mean difference (95% CI)	P-value
LDL	Normal	-0.085 (-0.188, 0.017)	0.138 (0.039, 0.237)	-0.223 (-0.366, -0.081)	0.002
	Marginal	-0.201 (-0.35, -0.052)	0.046 (-0.097, 0.188)	-0.247 (-0.455, -0.038)	0.021
	High	-0.393 (-0.639, -0.146)	0.179 (-0.118, 0.476)	-0.572 (-0.96, -0.184)	0.006
HDL	Normal	-0.069 (-0.107, -0.03)	-0.096 (-0.133, -0.059)	0.027 (-0.026, 0.081)	0.317
	Marginal	-0.036 (-0.085, 0.014)	-0.032 (-0.08, 0.015)	-0.003 (-0.072, 0.066)	0.922
	High	-0.040 (-0.094, 0.014)	-0.031 (-0.096, 0.034)	-0.009 (-0.094, 0.075)	0.823
Total cholesterol	Normal	-0.177 (-0.286, -0.067)	0.042 (-0.064, 0.148)	-0.219 (-0.371, -0.066)	0.005
	Marginal	-0.277 (-0.426, -0.129)	0.015 (-0.127, 0.157)	-0.293 (-0.499, -0.086)	0.006
	High	-0.424 (-0.757, -0.09)	-0.042 (-0.444, 0.36)	-0.382 (-0.909, 0.145)	0.146
Triglycerides	Normal	0.082 (-0.027, 0.191)	0.125 (0.02, 0.23)	-0.043 (-0.194, 0.108)	0.574
	Marginal	-0.032 (-0.171, 0.108)	0.063 (-0.07, 0.196)	-0.095 (-0.289, 0.099)	0.332
	High	0.186 (-0.24, 0.612)	0.023 (-0.489, 0.535)	0.163 (-0.503, 0.829)	0.615

Baseline value was adjusted in the model

Normal LDL-levels: Baseline LDL-C ≤3.36 mmol/L

Marginal LDL-levels: Baseline LDL-C 3.37-4.14 mmol/L

High LDL-levels: Baseline LDL-C >4.14 mmol/L
